# Dermoscopic Features of Plantar and Palmar Melanocytic Nevi in Children: Dermoscopy of Children's Plantar and Palmar Melanocytic Nevi

**DOI:** 10.1111/srt.70350

**Published:** 2026-04-16

**Authors:** Zixing Cui, Jianping Wu, Ling Fang, Chuanli Ren

**Affiliations:** ^1^ Department of Laboratory Medicine Clinical College of Yangzhou University Yangzhou Jiangsu China; ^2^ Xishan People's Hospital of Wuxi City Wuxi Branch of Zhongda Hospital Southeast University Wuxi Jiangsu China; ^3^ Department of Dermatology Anhui Provincial Children's Hospital Hefei China; ^4^ Department of Laboratory Medicine Northern Jiangsu People's Hospital Affiliated to Yangzhou University Yangzhou China

**Keywords:** dermoscopy pattern, palmoplantar nevus, pediatric dermatology

## Abstract

**Introduction:**

Plantar and palmar melanocytic nevi constitute a distinct subtype of nevi, characterized by unique dermoscopic patterns. Currently, limited research has focused on the dermoscopic features of plantar and palmar melanocytic nevi in children.

**Objective:**

To characterize the dermoscopic features of congenital and acquired plantar and palmar melanocytic nevus in children and to evaluate the diagnostic contribution of dermoscopy.

**Methods:**

A retrospective analysis was conducted on 180 pediatric patients initially clinically diagnosed with plantar and palmar nevi. Dermoscopic evaluation was performed using Dermo‐II system, followed by histopathological confirmation.

**Results:**

Histopathology confirmed 172 plantar and palmar nevi and 8 cases of black heel. The most prevalent dermoscopic patterns were the parallel furrow pattern (54.65%), fibrillar pattern (22.09%), and reticular pattern (10.47%). No significant differences were observed between congenital and acquired nevi. Dermoscopy achieved 100% concordance with histopathology, significantly higher than clinical diagnosis alone (95.56%; *p* = 0.0075).

**Conclusion:**

Dermoscopy is a reliable, non‐invasive adjunct for diagnosing pediatric plantar and palmar nevi, potentially reducing unnecessary excisions.

## 1 Introduction

Nevi, also referred to as pigmented nevi, are benign cutaneous tumors composed of clusters of melanocytes [1]. They can occur at any age and in any location on the skin or mucous membranes, with their number increasing with age [2, 3]. Studies indicate that the palms and soles are the most common sites of malignant melanocytic tumors in the Chinese population [4]. For the fear of the poor prognosis of malignant melanoma, despite the doctors' reassurance that pediatric plantar and palmar melanoma is exceedingly rare, parents of children with plantar and palmar nevi frequently report heightened anxiety, especially when the nevus undergoes enlargement, darkens in color, or exhibits an indistinct border, and they often seek surgical excision. This not only puts pressure on medical resources but may also expose children to unnecessary surgical risks. Distinguishing malignant melanocytic neoplasms from benign plantar and palmar melanocytic nevi remains a pivotal yet formidable challenge in pediatric patients. Traditionally, diagnosis relied on macroscopic observation and the ABCDE criteria [5], methods often inadequate for palmoplantar regions due to their unique anatomical structure and pigment distribution [6, 7].

Dermoscopy, a non‐invasive diagnostic tool, has proven useful in the early detection of malignant melanocytic tumors [8, 9].Melanocytic nevi on the palms and soles in children are frequently excised because of parental anxiety. The unique anatomy of these sites limits the accuracy of the conventional ABCDE rule. Dermoscopy, a non‐invasive technique, can improve diagnostic precision and reduce unnecessary surgery. We aimed to delineate the dermoscopic patterns of pediatric palmoplantar melanocytic nevi in Chinese children and to validate these findings against histopathology.

## 2 Data and Methods

### 2.1 General Information

From June 2021 to September 2024, 180 children (92 males and 88 females) initially diagnosed with plantar and palmar melanocytic nevi by the Departments of Dermatology at Xishan People's Hospital and Anhui Provincial Children's Hospital were consecutively enrolled. All participants underwent dermoscopy under the informed consent of their family members. The skin lesions were completely excised with a 0.1 cm clinical margin and were histopathologically confirmed as melanocytic nevus or black heel.

**Inclusion criteria**: (1) The initial clinical diagnosis of melanocytic nevus was independently rendered by two board‐certified dermatologists with senior academic rank, regardless of patient gender. (2) Lesions located in the palmoplantar regions (including the flexural sides of fingers and toes, but excluding the nails). (3) Patients under 18 years with histopathological confirmed melanocytic nevus or black heel.

**Exclusion criteria**: (1) Patients with other skin lesions or dermatological conditions at the lesion site. (2) Patients with metastatic malignant lesions in the palmoplantar regions. (3) Patients or their families who declined to participate.

### 2.2 Devices and Methods

#### 2.2.1 Equipment

Dermoscopy was performed using the Dermo‐II system (Beijing Demet Jikang Technology Development Co., Ltd., Beijing, China), with a magnification of 20×. The system was connected to a Canon EOS 750D digital camera.

#### 2.2.2 Examination Procedure

Parents were informed of the study's purpose, methods, and procedures prior to the examination. During dermoscopy, patients were positioned comfortably, ensuring the skin lesions were fully exposed and held flat. Representative clinical and dermoscopic images of each lesion were obtained, with descriptions of the microscopic features.

#### 2.2.3 Dermoscopic Image Diagnosis

Dermoscopy images were analyzed by two dermatologists experienced in dermoscopic diagnosis, in conjunction with an intelligent diagnostic system.

The dermoscopic patterns evaluated included: (1) Furrow parallel pattern. (2) Fibrous pattern. (3) Reticular pattern. (4) Mixed pattern (defined by the coexistence of two or more patterns). (5) Globular pattern. (6) Homogeneous pattern. (7) The presence of any of these patterns was diagnostic of melanocytic nevus.

The dermoscopic features of black heel disease are black patches, no pigment network, and no clear vascular structure, pebble‐like appearance on the spine [10].

#### 2.2.4 Histopathological Diagnosis

Surgical resection was performed on the palmoplantar skin lesions. The excised specimens were processed for fixation, embedding, sectioning, and histopathological examination by two dermatopathologists.

#### 2.2.5 Statistical Methods

Clinical and dermoscopic diagnoses were retrospectively compared with histopathological findings to calculate diagnostic concordance rates. Fisher's Exact Test analyses were conducted using SPSS 27.0 software. Differences in concordance rates were assessed using the chi‐square test, with p‐values < 0.05 considered statistically significant.

## 3 Results

### 3.1 Comparison Between Clinical Diagnosis and Histopathological Diagnosis

We first compared the initial clinical diagnosis of plantar and palmar melanocytic nevi with the corresponding histopathologic confirmation in 180 patients who completed both evaluations. Among them, 172 cases were plantar and palmar melanocytic nevi and 8 cases were black heel, resulting in a concordance rate of 95.56%. In contrast, the comparison between dermoscopic diagnosis and histopathological diagnosis revealed that 180 lesions were concordant, yielding a concordance rate of 100%. A Fisher's Exact Test (OR = 0.06) was used to compare the concordance rates of clinical diagnosis and dermoscopic diagnosis, and the difference was statistically significant (*p* = 0.0075). Detailed comparisons of concordance rates between clinical and dermoscopic diagnoses are provided in Table 1.

**TABLE 1:** Concordance rates between clinical and dermoscopic diagnoses compared with histopathology.

Diagnosis	Clinical diagnosis	Dermoscopy	OR	*p*
**Nevus**	180	172	—	—
**Black Heel**	0	8	—	—
**Conformity**	172 (95.56%)	180 (100.00%)	—	—
**Inconformity**	8 (4.44%)	0 (0.00%)	0.06	0.0075

### 3.2 Dermatoscopic Findings

Subsequently, we conducted a statistical analysis on 172 samples (58 congenital and 114 acquired)whose clinical pathological diagnosis was consistent with the dermoscopy diagnosis.

**FIGURE 1:**
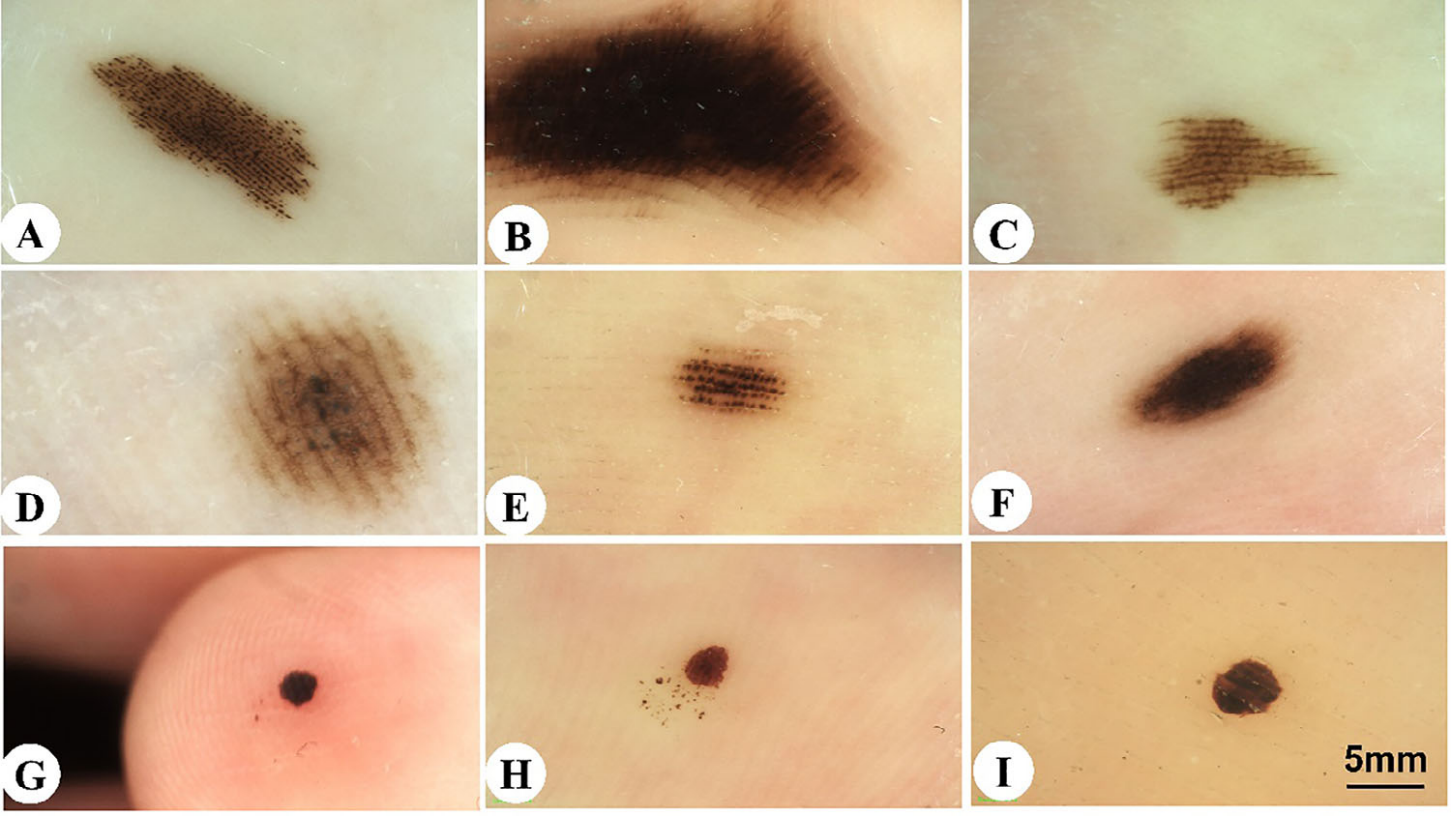
Dermoscopic patterns of plantar and palmar melanocytic nevi and black heel. (A) Parallel furrow. (B) Fibrillar. (C) Reticular. (D) Mixed. (E) Globular. (F) Homogeneous. (G–I) Black heel: Black patches, without pigment networks and without distinct vascular structures. Magnification: 20×, Scale bar: 5 mm.

The skin lesions of melanocytic nevus on the palms and soles were evaluated using dermatoscopy, revealing the following morphological features: (1) Parallel furrow pattern: The most common feature, observed in 94 cases (54.65%), was characterized by linear pigmentation distributed along the skin furrows (Figure 1A). (2) Fibrous pattern: This was the second most common pattern, observed in 38 cases (22.09%). It consisted of numerous fine, parallel fibrous pigments oriented either perpendicular or oblique to the skin furrows and ridges (Figure 1B). (3) Mesh‐like pattern: Found in 18 cases (10.47%), this pattern resembled the parallel pattern of the stratum corneum but displayed pigmented bands that connected or crossed adjacent cuticles (Figure 1C). (4) Mixed pattern: Identified in 14 cases (8.14%), this pattern exhibited two or more morphological modes. Among mixed patterns, the most common combination was the furrow parallel pattern with the globular pattern, which often presented as a “pea pod” appearance (Figure 1D). (5) Globular pattern: Observed in 4 cases (2.33%), this pattern consisted of pigment spheres of varying sizes corresponding to nevus cell nests (Figure 1E). (6) Homogeneous pattern: Detected in 4 cases (2.33%), this pattern exhibited uniform tan or brown regions distributed across both sulci and ridges (Figure 1F). Figure G‐I shows the feature of the black heel. Among all the melanocytic nevus lesions included in the study, none of the melanocytic nevi exhibited dermoscopic features suggestive of malignancy, such as atypical pigment networks, negative pigment networks, irregular streaks (e.g., radial streaming or pseudopodia), or irregular blue‐white veils commonly associated with malignant melanocytic tumors. A total of 172 cases were included (88 males and 84 females), and the distribution of these six dermoscopic modalities in both sexes is detailed in Table 2.

**TABLE 2:** Distribution of predominant dermoscopic pattern regarding gender.

Predominant pattern	Congenital			Acquired			Total (%)
	Total (%)	Male (%)	Female (%)	Total (%)	Male (%)	Female (%)	
Parallel furrow	35 (20.35)	19 (11.05)	16 (9.30)	59 (34.30)	30 (17.44)	29 (16.86)	94 (54.65)
Fibrillar	6 (3.49)	3 (1.74)	3 (1.74)	32 (18.60)	15 (8.72)	17 (9.88)	38 (22.09)
Reticular	8 (4.65)	4 (2.33)	4 (2.33)	10 (5.81)	5 (2.91)	5 (2.91)	18 (10.47)
Mixed	6 (3.49)	3 (1.74)	3 (1.74)	8 (4.65)	5 (2.91)	3 (1.74)	14 (8.14)
Globular	1 (0.58)	0 (0.00)	1 (0.58)	3 (1.74)	2 (1.16)	1 (0.58)	4 (2.33)
Homogeneous	2 (1.16)	1 (0.58)	1 (0.58)	2 (1.16)	1 (0.58)	1 (0.58)	4 (2.33)
**Total**	58	30	28	114	58	56	172

### 3.3 Histopathological Diagnosis

**FIGURE 2:**
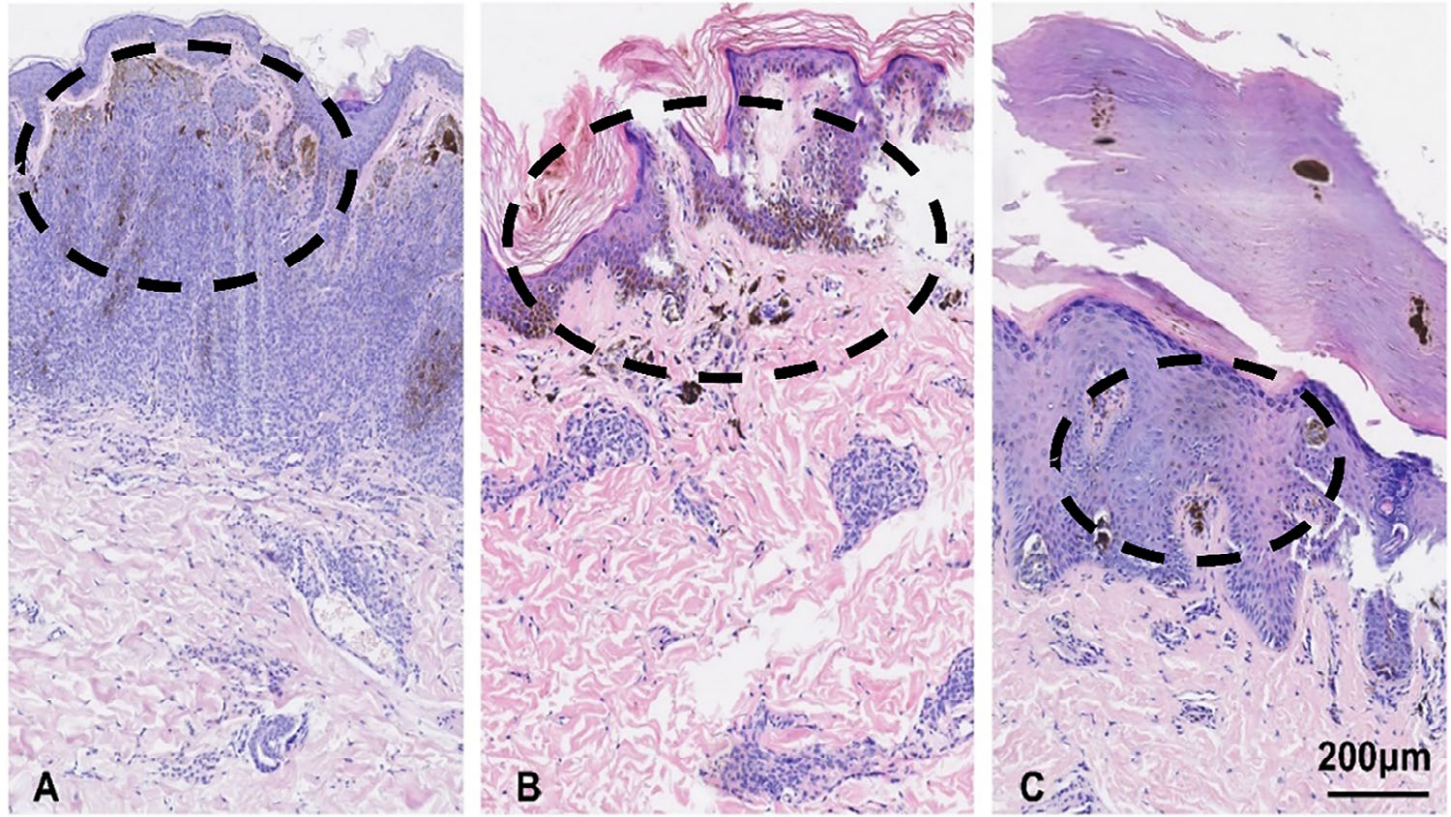
Histopathological features of melanocytic nevus. (A) Intradermal nevus: Proliferative melanocytes are localized within the dermis, with a distinct normal area between the melanocyte nests and the epidermis. (B) Compound nevus: Melanocyte nests are distributed in both the dermis and at the dermo‐epidermal junction. (C) Junctional nevus: Melanocytes are primarily located at the dermo‐epidermal junction, forming multiple nests with well‐defined boundaries. Magnification: 10×, Scale bar: 200 µm.

Histopathology is widely regarded as the “gold standard” for diagnosing melanocytic nevus. Based on their location within the skin, melanocytic nevus is classified into three types: junctional nevus, intradermal nevus, and compound nevus. The primary histopathological features of these types are as follows: (1) Melanocyte nests were absent in the epidermis, while proliferative melanocytes were observed in the dermis. Notably, a distinct normal area was often present between the melanocyte nests and the epidermis (Figure 2A). (2) Melanocyte nests were identified both in the dermis and at the dermo‐epidermal junction (Figure 2B). (3) Small melanocyte nests were observed at the superficial dermis and the dermo‐epidermal junction. These nests were predominantly distributed along the dermo‐epidermal junction beneath the cuticular ridges (Figure 2C).

## 4 Discussion

Melanocytic nevus is a common clinical benign lesion that can manifest in various anatomical regions and at different ages. Melanocytic nevus located in the palmar and plantar areas require differentiation from malignant melanoma, pigmented basal cell carcinoma, and other malignant tumors [11]. Misdiagnosis rates are notably high when skin lesions exhibit atypical features. Furthermore, the distribution of dermoscopic patterns in plantar and palmar nevi may be influenced by anatomical location and mechanical pressure. Previous studies have demonstrated that weight‐bearing areas, such as the heel and metatarsal heads, tend to exhibit fibrillar patterns more frequently due to continuous mechanical stress and altered skin surface scanning. In contrast, non‐weight‐bearing regions, such as the foot arch, more commonly display lattice‐like patterns. These observations suggest that pressure‐related factors and anatomical variations contribute to the heterogeneity of dermoscopic presentations in palmoplantar melanocytic nevi. Additionally, the risk of malignant transformation of melanocytic nevi in friction‐prone areas of the palms and soles is elevated [12]. Consequently, histopathological examination is often necessary to establish a definitive diagnosis. In pediatric patients, invasive procedures such as biopsy and pathological examination are typically avoided due to the young age of the patients, as blind surgical excision could result in unnecessary trauma. Dermoscopy offers a non‐invasive, real‐time, and user‐friendly diagnostic modality. Serving as a bridge between macroscopic diagnosis and histopathological assessment, dermoscopy has the potential to minimize unnecessary biopsies and indiscriminate excisions, thereby enhancing diagnostic accuracy. To our knowledge, this is the largest study to systematically characterize the dermoscopic patterns of palmoplantar melanocytic nevi in Chinese children. Dermoscopy increased diagnostic accuracy from 95.56% to 100%, underscoring its value as a tool to avoid unnecessary excision in this age group.

Currently, there's a lack of studies on the dermatoscopic and pathological features of plantar and palmar melanocytic nevi in Asian children. As palmar‐plantar regions are common sites for melanoma in Asian and children's melanoma features differ from adults, large sample comparative observations become crucial. Childhood represents a dynamic period of change during which melanocytic nevus appears and grows. Differentiating melanocytic nevus from malignant melanoma has consistently been a diagnostic challenge [13]. This difficulty arises not only because certain pigmented nevus exhibits atypical clinical morphology and histopathological features resembling melanoma but also due to the complex relationship between the two conditions. Some malignant melanomas may originate from congenital or acquired benign melanocytic nevus or develop from dysplastic nevus [6, 11, 14]. Moreover, the dermoscopic features of palmar‐plantar melanocytic nevus in children can change over time due to age and external stimuli [3]. For instance, a transition from the sulcus parallel pattern to the fibrous pattern may occur, accompanied by alterations in pigmentation intensity. Historically, surgical excision of palmar‐plantar melanocytic nevus, including those in children, has been a common treatment approach. However, in a study involving 493 children who underwent surgical excision of melanocytic lesions, only three cases were identified as malignant melanoma [15]. Considering the young age of pediatric patients, the poor compliance with local anesthesia, and the potential risks associated with general anesthesia, we advise against indiscriminate surgical excision of palmar‐plantar melanocytic nevus in young children. Instead, we advocate for regular clinical and dermoscopic monitoring. Furthermore, recent studies on skin of color dermoscopy have provided important insights into pattern variations across different ethnicities. As reported by Karampinis [16] in their comprehensive review of pediatric dermatology in skin of color, volar skin in darker‐skinned children may exhibit pigment networks or dot‐like distributions due to physiological variations in melanin distribution. It is worth noting that nevi of different skin colors and different anatomical regions may vary, and their dermoscopic patterns also differ. Although our study primarily included children with Fitzpatrick skin types III‐IV, we acknowledge that in dark skin types (including some individuals in the Asian cohort), the contrast of pigmentation may affect the visualization of certain patterns. This comparison emphasizes the need to consider age and skin type factors in dermoscopy assessment. Currently, there have been no large‐scale reports on the dermoscopic patterns of pigmented nevi on the palms and soles of children of different skin types. Nevi of different skin colors and different anatomical regions may vary, and their dermoscopic patterns also differ. While Mernissi et al. (2025) demonstrated significant correlations between dermoscopic patterns and anatomical locations (palmar vs. sole) in mixed‐age populations, such differences appear less pronounced in pediatric cohorts [17]. Currently, there have been no large‐scale comparative studies specifically examining palmar versus sole nevi across different pediatric ethnic populations. Future multi‐center collaborative studies with larger pediatric sample sizes are warranted to elucidate these potential age‐ and ethnicity‐related variations.

Dermoscopy is widely recognized as a valuable tool for the noninvasive diagnosis of melanocytic lesions and for the early detection of malignant melanocytic tumors. The unique dermoscopic patterns of the palmar and plantar regions are primarily attributed to the thick, hairless skin and the distinct furrow and ridge structures. These features result in melanocytic nevus predominantly located in the epidermal processes of the boundary ridges or in melanin transfer through epidermal melanin units to keratinocytes near the furrows, producing diverse dermoscopic patterns. These patterns often manifest as pigment distributions along furrows and ridges, forming grid or fibrous patterns. Our study found that there was no statistically significant difference in the dermoscopic features of congenital and acquired nevus on the palms and soles of children. The most common feature was the parallel striation pattern, followed by the fibrous pattern and the grid pattern. The concordance rate between clinical diagnosis and histopathological diagnosis was 95.56%, while the concordance rate between dermoscopic diagnosis and histopathological diagnosis was 100.00%.

The distinctive dermoscopic models of palmar‐plantar melanocytic nevus were first described by Saida in 1995, which remains the most common pattern observed across Asian ethnicities [18], and subsequently refined by Malvehy and Puig in 2004, who proposed additional patterns, including the network, globular, and homogeneous models [19]. At present, the research on the dermoscopic features of plantar and palmar melanocytic nevi of children is very limited [12, 20]. Our study provides a more detailed and comprehensive characterization, including mixed, globular, and homogeneous patterns. Our findings are consistent with previous studies conducted in Asian populations. Similarly, studies from Korea have reported comparable distributions of dermoscopic patterns in pediatric and adult populations with palmoplantar nevi [21, 22]. Our dermoscopic findings align closely with these studies. The frequency of the parallel furrow pattern (54.65%) aligns closely with Korean data (51.7%) [21]. Furthermore, in acquired melanocytic nevi, the frequency of the parallel furrow pattern (34.3%) is highly consistent with the corresponding studies conducted in South Korea (37.5%) and Japan (34.6%) [12, 22]. Notably, the parallel ridge pattern, highly specific for melanoma, was absent in our benign cases, consistent with Asian literature [23]. These comparisons support the generalizability of our findings to Asian populations and highlight consistent patterns across different Asian ethnicities. Nonetheless, the present study has limitations, including the absence of histological correlation and a limited small sample size. Generally, congenital melanocytic nevi are believed to carry a higher malignant potential than acquired nevi, our study did not identify dermoscopic features that could reliably distinguish between the two entities. This study was limited by its retrospective design and lack of long‐term follow‐up data. Additionally, the absence of melanoma cases precluded assessment of dermoscopic sensitivity for malignancy detection. Future research involving larger, randomized, and long‐term studies is necessary to validate these findings.

The statistical results of this study demonstrate that the diagnostic accuracy of dermoscopy is significantly higher than that of clinical visual assessment. These findings reinforce confidence in utilizing dermoscopy to evaluate melanocytic nevus, thereby reducing the need for invasive diagnostic procedures. Nevertheless, analysis of misdiagnosed cases highlights the need for differentiation between melanocytic nevus and other conditions, such as melanocytomas, viral warts, and black heel. Histopathological examination remains the gold standard when dermoscopic findings are inconclusive.

For pediatric patients with melanocytic nevus in the palmar and plantar regions, the higher diagnostic accuracy provided by dermoscopy offers a solid foundation for treatment decisions. Particularly for children unable to undergo invasive histopathological examinations, a definitive benign diagnosis enables the selection of appropriate treatment options, including elective surgery or observation. Consequently, dermoscopy is affirmed as a safe, convenient, and effective method for the clinical diagnosis of melanocytic nevus.

## Ethics Statement

The research was approved by the Ethics Committee of Xishan People's Hospital of Wuxi City, ethical approval number is xs2024ky079.

## Conflicts of Interest

The authors declare no conflicts of interest.

## Data Availability

The data supporting the findings of this study are available from the corresponding author upon request. These data are not publicly accessible due to privacy and ethical considerations.
